# Metabolic Rewiring of Bacterial Pathogens in Response to Antibiotic Pressure—A Molecular Perspective

**DOI:** 10.3390/ijms26125574

**Published:** 2025-06-11

**Authors:** Carlo Acierno, Fannia Barletta, Riccardo Nevola, Luca Rinaldi, Ferdinando Carlo Sasso, Luigi Elio Adinolfi, Alfredo Caturano

**Affiliations:** 1 Department of Infectious Diseases, San Carlo Hospital, 85100 Potenza, Italy; 2 Department of Anesthesiology and Intensive Care, San Carlo Hospital, 85100 Potenza, Italy; fannia.barletta@ospedalesancarlo.it; 3 Liver Unit, AORN S. G. Moscati, “A. Landolfi” Hospital, 83029 Solofra, Italy; riccardo.nevola@unicampania.it; 4 Department of Medicine and Health Science “Vincenzo Tiberio”, Università degli Studi del Molise, 86100 Campobasso, Italy; luca.rinaldi@unimol.it; 5 Department of Advanced Medical and Surgical Sciences, University of Campania “Luigi Vanvitelli”, 80138 Napoli, Italy; ferdinandocarlo.sasso@unicampania.it (F.C.S.); luigielio.adinolfi@unicampania.it (L.E.A.); 6 Department of Human Sciences and Promotion of the Quality of Life, San Raffaele Roma Open University, 00166 Rome, Italy; alfredo.caturano@uniroma5.it

**Keywords:** metabolic rewiring, antibiotic tolerance, bacterial persistence, redox metabolism, glyoxylate cycle, membrane adaptation, metabolic adjuvants, antibiotic resistance, host–pathogen interaction, quorum sensing

## Abstract

Antibiotic pressure exerts profound effects on bacterial physiology, not limited to classical genetic resistance mechanisms. Increasing evidence highlights the ability of pathogens to undergo metabolic rewiring—an adaptive, reversible reorganization of core metabolic pathways that promotes survival under antimicrobial stress. This review provides a comprehensive analysis of antibiotic-induced metabolic adaptations, encompassing glycolysis, the tricarboxylic acid cycle, fermentation, redox balance, amino acid catabolism, and membrane biosynthesis. We critically examine how diverse antibiotic classes—including β-lactams, aminoglycosides, quinolones, glycopeptides, polymyxins, and antimetabolites—interact with bacterial metabolism to induce tolerance and persistence, often preceding stable resistance mutations. In parallel, we explore the ecological and host-derived signals—such as immunometabolites and quorum sensing—that modulate these metabolic responses. Therapeutically, targeting metabolic pathways offers promising strategies to potentiate antibiotic efficacy, including enzyme inhibition, metabolic adjuvants, and precision-guided therapy based on pathogen metabolic profiling. By framing metabolic plasticity as a dynamic and evolutionarily relevant phenomenon, this review proposes a unifying model linking transient tolerance to stable resistance. Integrating metabolic rewiring into antimicrobial research, clinical diagnostics, and therapeutic design represents a necessary paradigm shift in combating bacterial persistence and resistance.

## 1. Introduction

Amid the ongoing antimicrobial resistance (AMR) crisis, bacterial survival strategies in response to antibiotic pressure have become a topic of increasing scientific and clinical relevance.

Historically, research has focused primarily on genetic mechanisms of resistance, such as mutations in molecular targets, the overexpression of efflux pumps, enzymatic modifications, and horizontal gene transfer [1,2,3].

In recent years, however, a new dimension of bacterial resistance has emerged—more dynamic and plastic—centered on the ability of pathogens to profoundly reconfigure their metabolic architecture to adapt to hostile conditions, including those imposed by sublethal antibiotic exposure.

This phenomenon, known as “metabolic rewiring,” involves the reorganization of major metabolic pathways to reduce drug susceptibility, optimize energy production under stress, and modulate the expression of virulence and persistence genes [4,5].

Selective pressure from antibiotics acts not only as a mutagenic force but also as a biochemical stimulus capable of inducing reversible alterations in redox balance, energy homeostasis, and macromolecular biosynthesis [6].

An increasing number of studies have shown that metabolic adaptation can affect antibiotic susceptibility even in the absence of canonical genetic changes, through the modulation of pathways such as glycolysis, the tricarboxylic acid (TCA) cycle, the glyoxylate shunt, lipid metabolism, and oxidative stress responses [7,8].

This phenotypic flexibility enables bacteria to enter a state of low metabolic activity—known as persistence—in which antibiotic efficacy is drastically diminished [9].

An additional layer of complexity arises from the interaction between bacterial metabolism and the host environment, including immune signals, nutrients, commensal microbiota, and temperate phages. In particular, the ability of bacteria to integrate environmental cues via quorum sensing (QS) systems and central metabolic pathways enables a coordinated adaptive response that often determines the outcome of infection [10].

Despite the emerging significance of these phenomena, current reviews in the literature tend to address metabolic rewiring only partially, often focusing on individual pathways or collateral effects of antibiotic pressure, without integrating their systemic, evolutionary, and clinical implications.

In particular, there is a lack of critical syntheses that connect metabolic alterations to the molecular mechanisms of tolerance and the subsequent emergence of stable resistance mutations, as highlighted by recent experimental and conceptual models.

This review aims to fill that gap by offering a critical and systemic overview of metabolic reprogramming in bacterial pathogens in response to antibiotic pressure.

Specifically, it seeks to:

- analyze the main metabolic circuits involved in rewiring induced by antibiotics from different classes (β-lactams, aminoglycosides, quinolones, glycopeptides, etc.);
- describe the interplay between molecular signaling, redox responses, and mutagenesis;
- discuss the evolutionary and therapeutic implications of these mechanisms, clearly distinguishing between metabolic plasticity and acquired genetic resistance; and
- propose an integrated conceptual framework for identifying novel metabolic targets, with a view toward metabolite-guided antibiotic therapy.

## 2. Key Concepts: Operational Definition of Metabolic Rewiring and Distinction from Genetic Resistance

The concept of metabolic rewiring refers to the set of functional and dynamic restructuring processes of an organism’s metabolic networks in response to environmental perturbations, including antibiotic pressure [11].

In bacteria, such reprogramming constitutes a non-genetic, reversible, and often transient strategy aimed at optimizing bioenergetic resources and ensuring survival under hostile conditions, through the remodeling of key pathways such as glycolysis, the Krebs cycle, oxidative phosphorylation, lipid biosynthesis, and amino acid metabolism [12,13,14].

It is essential to distinguish this phenomenon from classical genetic resistance, which involves stable and heritable modifications in the bacterial genome [15].

**Figure 1:**
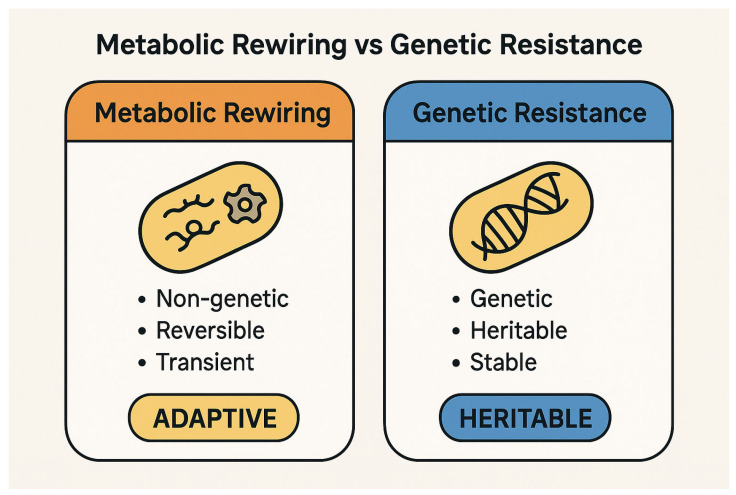
Comparative schematic of metabolic rewiring and genetic resistance in bacterial adaptation to antibiotic pressure. Metabolic rewiring is characterized by non-genetic, reversible, and transient changes that enable adaptive responses to environmental stress. In contrast, genetic resistance results from stable, heritable genetic modifications, such as mutations or horizontal gene transfer, that persist even in the absence of selective pressure. While metabolic rewiring supports immediate survival under stress, it may serve as a precursor to stable resistance by increasing the likelihood of mutation fixation. Image created using BioRender (accessed via web application, version as of May 2025), GraphPad Prism (version 10.1.2-2024), and PowerPoint (Microsoft 365, version 2404).

The conceptual distinction between metabolic rewiring and genetic resistance is fundamental to understanding bacterial adaptation under antibiotic stress. While the former represents a dynamic, reversible survival strategy, the latter entails stable genetic changes that confer heritable resistance (Figure 1).

These modifications include point mutations in antibiotic targets, acquisition of resistance genes via plasmids or transposons, and constitutive expression of inactivating enzymes or efflux pumps [16,17].

Metabolic rewiring represents an adaptive phenotypic response, at times overlapping with the phenomenon of “tolerance,” in which bacteria temporarily survive antibiotic exposure without a corresponding change in the Minimum Inhibitory Concentration (MIC) [18,19].

This state of reduced metabolic activity—typically associated with diminished reactive oxygen species (ROS) production, low respiration, and accumulation of toxic intermediates—has been shown to promote survival even in the absence of genetic alterations, as demonstrated in *Escherichia coli* and *Staphylococcus aureus* [20,21,22].

Several studies have indicated that, under sublethal antibiotic pressure, bacteria can redirect carbon flux toward secondary pathways—such as the glyoxylate cycle—thereby reducing ROS generation and oxidative damage induced by quinolones or aminoglycosides [5,7,23].

This results in a transient form of metabolic resistance, which may nonetheless serve as a pre-mutational platform, facilitating the selection of bona fide genetic resistance events [24].

**Figure 2:**
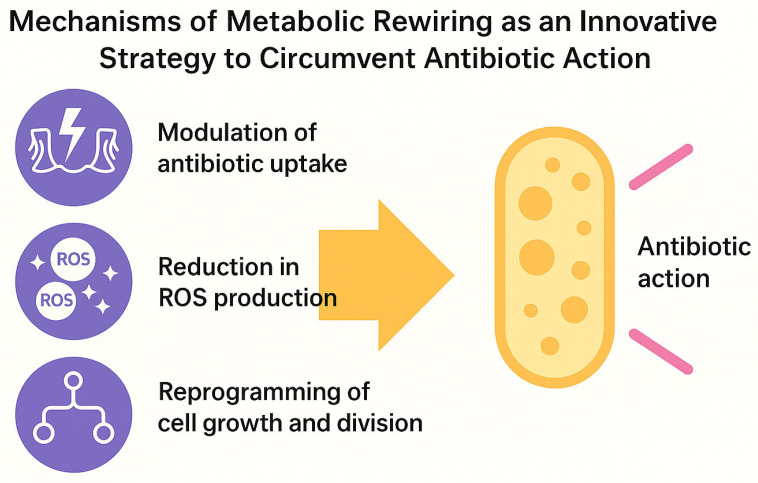
Mechanistic overview of how metabolic rewiring enables bacteria to circumvent antibiotic action. Key adaptive strategies include (1) the modulation of antibiotic uptake via changes in membrane composition or electrochemical gradients; (2) the reduction of intracellular reactive oxygen species (ROS) production by suppressing oxidative metabolism; and (3) the reprogramming of cell growth and division to delay or escape the temporal window of antibiotic efficacy. These processes contribute to the establishment of a transient, drug-tolerant phenotype, facilitating bacterial persistence under antimicrobial stress. Image created using BioRender (accessed via web application, version as of May 2025), GraphPad Prism (version 10.1.2-2024), and PowerPoint (Microsoft 365, version 2404).

These mechanisms—the modulation of antibiotic uptake, the reduction in ROS production, and growth reprogramming—collectively define the functional basis of metabolic rewiring and its role in promoting bacterial tolerance (Figure 2).

At the molecular level, metabolic plasticity can interfere with antibiotic action through at least three mechanisms:

- Modulation of antibiotic uptake: changes in membrane potential or lipid composition can reduce the entry of cationic antibiotics [25,26].
- Reduction in ROS production: with significant consequences for the efficacy of bactericidal agents, whose activity depends on lethal oxidative stress [6,27].
- Reprogramming of growth and division: cyclic arrest or metabolic slowdown allows bacteria to outlast the temporal window of antibiotic action, mimicking a dormant state [22,28].

The principal molecular mechanisms underlying bacterial metabolic rewiring in response to antibiotics are summarized in Table 1.

It is also important to emphasize that metabolic rewiring is not merely a passive response: in many bacterial species, environmental signals (nutrients, stress molecules, low-dose antibiotics) can activate bona fide regulatory circuits that actively promote the transition to alternative metabolic states, via redox transducers, quorum sensing systems, and global regulators such as Crp, SoxR, and ArcA [29,30].

Finally, from an evolutionary perspective, an intriguing synergy emerges between metabolic plasticity and genetic selection: bacteria that survive through metabolic reprogramming may provide the phenotypic substrate within which stable mutations are subsequently selected, thus positioning metabolic rewiring as a potential precursor to genuine genetic resistance [31,32].

A comparative overview of the key features distinguishing metabolic rewiring from classical genetic resistance is provided in Table 2.

## 3. Metabolic Responses to Different Antibiotic Classes

The efficacy of antibiotics depends not only on their affinity for specific molecular targets but also on the metabolic state of the bacterial cell at the time of exposure [33].

Consequently, antibiotic pressure can induce distinctive, sometimes counterintuitive, metabolic responses that modulate drug effects without altering the MIC [7,34].

The following subsections examine how different classes of antibiotics interact with central metabolic pathways, sometimes promoting transient adaptations associated with tolerance, persistence, or evolutionary selection.

### 3.1. β-Lactams and Peptidoglycan Metabolism

β-lactam antibiotics act by inhibiting penicillin-binding proteins (PBPs), thereby blocking peptidoglycan synthesis and inducing osmotic lysis [35,36,37].

However, the activation of compensatory metabolic pathways may mitigate their efficacy.

In *Escherichia coli*, sublethal exposure to β-lactams is associated with increased expression of genes involved in the synthesis of peptidoglycan precursors, including UDP-N-acetylmuramyl. Concurrently, some evidence suggests a reorganization of carbohydrate metabolism, potentially aimed at supporting cell wall reconstruction under antibiotic stress [6,38,39].

The co-activation of the glyoxylate cycle suggests a carbon-conserving strategy that facilitates the regeneration of damaged cell walls [40,41].

In *Staphylococcus aureus* strains, some evidence indicates that downregulation of the tricarboxylic acid (TCA) cycle, accompanied by increased fermentative metabolic activity, may contribute to reduced production of reactive oxygen species (ROS) and a deceleration of cellular growth rate.

These metabolic adaptations have been linked to increased tolerance to β-lactam antibiotics, likely due to the reduced activation of cell death mechanisms dependent on oxidative stress and active replication [42,43].

### 3.2. Aminoglycosides and Redox Response

Aminoglycosides, including gentamicin and tobramycin, rely on an active uptake mechanism dependent on the membrane electrochemical gradient (proton motive force), which is required to cross the cytoplasmic membrane and reach their ribosomal target within the bacterial cell [44].

Under conditions that compromise this gradient—such as anoxia, medium acidification, or the concomitant use of respiratory chain inhibitors—the bactericidal efficacy of aminoglycosides may be significantly reduced [44,45].

Their effectiveness is therefore linked to the respiratory state of the cell.

Several studies have shown that metabolic downshifting toward fermentation or microaerophilic conditions induces functional tolerance to aminoglycosides, reducing mitochondrial ROS production and lethal cellular damage [6,7,12].

In *Pseudomonas aeruginosa*, the activation of compensatory redox pathways (e.g., glutathione biosynthesis, the NADPH pathway) is associated with a persistent phenotype tolerant to aminoglycosides, even in the absence of genetic resistance mechanisms [46].

### 3.3. Quinolones and Oxidative Stress

Quinolones, such as ciprofloxacin and levofloxacin, inhibit topoisomerases II and IV, resulting in double-strand DNA breaks [47,48].

However, their activity is amplified by extensive production of secondary ROS [49].

Metabolic modulation can thus interfere with the drug’s efficacy [50].

In particular, suppression of the TCA cycle and the respiratory chain reduces mitochondrial ROS accumulation, limiting the secondary damage induced by quinolones [6,7].

In *Mycobacterium tuberculosis* and *Salmonella enterica*, activation of the glyoxylate cycle represents a metabolic adaptation strategy under environmental and nutritional stress conditions [4,51,52].

Although direct experimental evidence linking this pathway to a reduced NADH pool or to the mitigation of antibiotic lethality mediated by ROS is not yet available, it is plausible that rerouting of metabolic flux through non-decarboxylating anaplerotic pathways may contribute to lower reactive oxygen species generation, thereby influencing sensitivity to quinolones and other bactericidal antibiotics [51,52].

### 3.4. Glycopeptides and Carbon Metabolism

Glycopeptides, such as vancomycin and teicoplanin, inhibit peptidoglycan polymerization by binding to D-Ala-D-Ala residues [53,54].

However, their effectiveness is diminished under conditions of slow growth or dormancy [12].

Progressive exposure to vancomycin induces a series of complex physiological and metabolic adaptations in *Staphylococcus aureus*, associated with the evolution toward an intermediate-resistant phenotype.

Metabolomic analyses have revealed extensive remodeling of intracellular metabolism consistent with an energy-saving strategy.

This reorientation includes changes in the profiles of over 200 metabolites, with reduced virulence, diminished adhesion capacity, and potential reorganization of anabolic and catabolic fluxes, although no specific activation of gluconeogenesis or direct inhibition of glycolysis or the Krebs cycle has been documented.

The observed alterations appear aimed at enhancing survival under antibiotic and immune stress, rather than supporting active growth [55].

In certain *Enterococcus faecalis* strains subjected to selective pressure from glycopeptides, an increase in the synthesis of cell wall precursors via alternative biosynthetic pathways has been observed, supported by a reorientation of carbon metabolism [56].

In particular, the activation of anaplerotic fluxes along the phosphoenolpyruvate–pyruvate–oxaloacetate axis suggests a functional adaptation aimed at ensuring the availability of intermediates for muramyl biosynthesis under conditions of vancomycin-mediated inhibition of conventional peptidoglycan synthesis [56].

Such metabolic remodeling may represent an ancillary mechanism supporting non-van gene-mediated resistance.

### 3.5. Polymyxins and Phospholipids

Polymyxins (e.g., colistin) bind to lipopolysaccharides on the outer membrane of Gram-negative bacteria, causing lipid disorganization [57,58].

In response to this selective pressure, several Gram-negative pathogens activate two-component regulatory systems (PmrA/PmrB and PhoP/PhoQ), which promote Lipid A modification through the addition of cationic groups such as 4-amino-4-deoxy-L-arabinose (L-Ara4N) and phosphoethanolamine (pEtN), catalyzed by the enzymes ArnT and EptA, respectively [59].

These modifications reduce the net negative charge of the outer membrane, hindering electrostatic interactions with polymyxins.

Concomitantly, alterations in membrane lipid composition have been observed, including a reduction in unsaturated fatty acids and a restructuring of biosynthetic pathways involving acetyl-CoA, the glycolate pathway, and acyl carrier proteins (ACPs), suggesting a metabolic rewiring aimed at supporting lipid plasticity and antimicrobial tolerance [60].

In *Klebsiella pneumoniae* infections, resistance to polymyxins—particularly colistin—is mediated by a wide array of genetic adaptations that converge on altering outer membrane lipid structure and modulating two-component response systems. In particular, modifications of Lipid A through the addition of L-Ara4N or pEtN reduce antibiotic binding affinity [61,62].

These changes are frequently associated with inactivating mutations or insertions in the *mgrB* gene, as well as alterations in the regulatory systems PhoPQ, PmrAB, and, less commonly, CrrAB, leading to overexpression of the *pmrHFIJKLM* operon and the *phoP*, *phoQ*, *pmrK*, and *pmrC* genes [61,62,63].

In numerous clinical cohorts, the emergence of resistance has been more frequently associated with selective pressure from prior polymyxin exposure than with human-to-human transmission of resistant clones and is often linked to high MIC values (≥64 µg/mL) and high-risk epidemic clones such as ST11 and ST258 [62,63].

Notably, the insertion of mobile elements such as ISkpn14 into the *mgrB* gene represents one of the predominant mechanisms of gene inactivation in resistant strains isolated in China [61,62,63].

### 3.6. Antimetabolite Antibiotics: Direct Effect and Biosynthetic Compensation

Antimetabolites, such as sulfonamides, trimethoprim, and fosfomycin, exert their antibacterial activity by interfering with biosynthetic pathways essential for bacterial survival.

Specifically, sulfonamides and trimethoprim act by sequentially inhibiting enzymes in the folic acid synthesis pathway: the former block dihydropteroate synthase, while the latter inhibits dihydrofolate reductase, thereby impairing the production of thymidine and purine nucleotides required for DNA synthesis [64].

Fosfomycin, by contrast, inhibits an early and critical step in bacterial cell wall biosynthesis by irreversibly blocking the enzyme MurA (UDP-N-acetylglucosamine enolpyruvyl transferase), thus preventing the formation of muramyl precursors and resulting in rapid bacterial lysis [65].

The inhibitory effect of trimethoprim on the folate pathway can be partially circumvented by *E. coli* through the activation of adaptive metabolic circuits, including the uptake of exogenous purine bases and the modulation of de novo nucleotide synthesis [66].

This condition triggers adaptive transcriptional responses, including derepression of purine biosynthesis genes mediated by reduced activity of the PurR repressor, and indirectly, activation of the stringent response via RelA, leading to the synthesis of (p)ppGpp.

The combined effect of these mechanisms promotes the expression of alternative biosynthetic pathways aimed at restoring nucleotide homeostasis [67].

In *Escherichia coli*, inhibition of peptidoglycan synthesis by fosfomycin may induce adaptive metabolic responses, including modulation of the glucosamine-6-phosphate metabolism and activation of the pentose phosphate pathway, in order to support the biosynthesis of cellular precursors and the production of NADPH [68].

Table 3 provides a comprehensive synthesis of antibiotic-specific metabolic rewiring strategies that modulate bacterial survival and stress tolerance (Table 3).

## 4. Metabolic Pathways Involved in Rewiring: Glycolysis, TCA, Oxidative Stress, Amino Acid Metabolism, and Membrane Biosynthesis

Bacterial adaptive responses to antibiotics extend beyond growth modulation and entail a profound reprogramming of central metabolic circuits. This section examines the major biochemical nodes subjected to rewiring, highlighting their roles in tolerance, persistence, and the evolution of resistance.

### 4.1. Glycolysis and Fermentation: Alternative Fluxes and NAD^+^ Homeostasis

Glycolysis is a core pathway for rapid ATP production and the generation of metabolic intermediates [69].

Under stress conditions induced by bactericidal antibiotics, many bacteria reprogram the glycolytic metabolism by diverting pyruvate toward fermentative pathways rather than aerobic respiration. This metabolic reconfiguration contributes to reduced production of reactive oxygen species (ROS) associated with the electron transport chain, thereby preserving intracellular redox balance, particularly the NAD^+^/NADH ratio [70,71].

In *Staphylococcus aureus*, the inhibition of aerobic respiration during infection prompts a metabolic adaptation toward fermentation, characterized by the production of lactate and acetate [72].

This process facilitates regeneration of NAD^+^ from NADH, maintaining redox homeostasis and supporting ATP synthesis through substrate-level phosphorylation [73].

Such a bioenergetic adaptation is crucial for the survival of persister cells in hostile environments.

The transition from aerobic respiration to fermentation is modulated by environmental cues, such as oxygen and nutrient availability, and by specific regulatory systems, including SrrAB and NreBC [74,75].

These metabolic adaptations promote NAD^+^ regeneration and sustain ATP production via substrate-level phosphorylation, ensuring cellular survival under redox stress or limited oxygen supply [74,75].

Table 4 summarizes the main fermentative adaptations and glycolytic reprogramming strategies supporting bacterial survival under antibiotic-induced stress.

### 4.2. Krebs Cycle and Glyoxylate Shunt: Carbon-Sparing Survival

The tricarboxylic acid (TCA) cycle serves as a central hub for energy generation and biosynthetic precursors [76].

However, several of its enzymes—such as aconitase, succinate dehydrogenase, and α-ketoglutarate dehydrogenase—contain Fe–S clusters that are highly sensitive to oxidative stress [77].

During antibiotic exposure, bacteria may suppress or restructure the TCA cycle to limit the production of reactive oxygen species (ROS).

**Figure 3:**
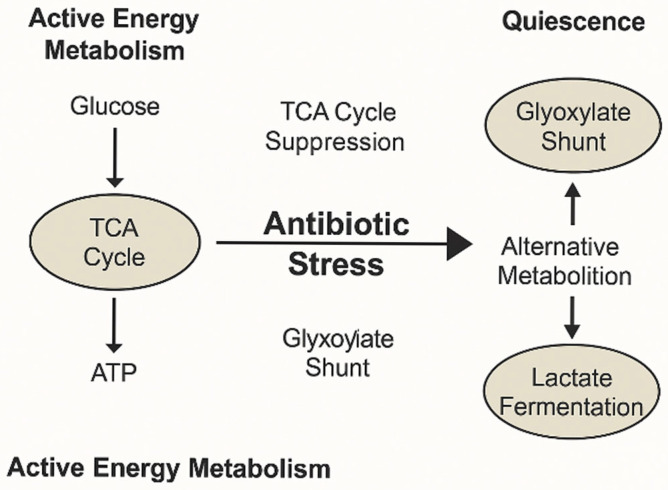
Metabolic adaptation of bacteria under antibiotic stress involves a transition from active energy metabolism, predominantly driven by the tricarboxylic acid (TCA) cycle, to alternative, low-energy states that support survival. Under normal conditions, glucose metabolism fuels the TCA cycle to generate ATP. Upon antibiotic exposure, suppression of the TCA cycle leads to redirection of metabolic flux through pathways such as the glyoxylate shunt and lactate fermentation. This shift facilitates the maintenance of redox balance and promotes a quiescent, drug-tolerant phenotype. These adaptations contribute to antibiotic tolerance and persistence by minimizing oxidative stress and metabolic activity. Image created using BioRender (accessed via web application, version as of May 2025), GraphPad Prism (version 10.1.2-2024), and PowerPoint (Microsoft 365, version 2404).

This metabolic shift is exemplified by the transition from TCA cycle-driven energy metabolism to alternative pathways, such as the glyoxylate shunt and lactate fermentation, which support bacterial quiescence and tolerance during antibiotic stress (Figure 3). These adaptations enable carbon conservation and the reduction of oxidative stress, contributing to survival under adverse conditions.

This metabolic modulation reduces intracellular oxidative stress, contributing to antibiotic tolerance and survival in hostile environments [78,79].

In response to oxidative stress or limited nutrient availability, many pathogenic bacteria activate the glyoxylate shunt, a variant of the TCA cycle [80].

This pathway, mediated by the enzymes isocitrate lyase and malate synthase, allows bypass of oxidative decarboxylation reactions, thus avoiding carbon loss in the form of CO_2_ [80].

This metabolic adaptation enables carbon conservation and the generation of essential biosynthetic intermediates, thereby supporting pathogen survival under hostile conditions and during host infection [81].

In *Mycobacterium tuberculosis*, activation of the glyoxylate cycle—catalyzed by isocitrate lyase and malate synthase—is essential for intracellular survival during the latent phase of infection [82,83].

This adaptation allows *M. tuberculosis* to evade the oxidative decarboxylation steps of the TCA cycle, preserving carbon and generating key intermediates for gluconeogenesis and macromolecular biosynthesis [84].

Central metabolic reconfiguration in *M. tuberculosis*, characterized by suppression of oxidative respiration and activation of the glyoxylate cycle, supports pathogen persistence in the hostile microenvironments typical of latent infection [85].

This adaptation limits intracellular ROS generation, contributing to drug tolerance and survival in the presence of bactericidal antibiotics [82].

**Figure 4:**
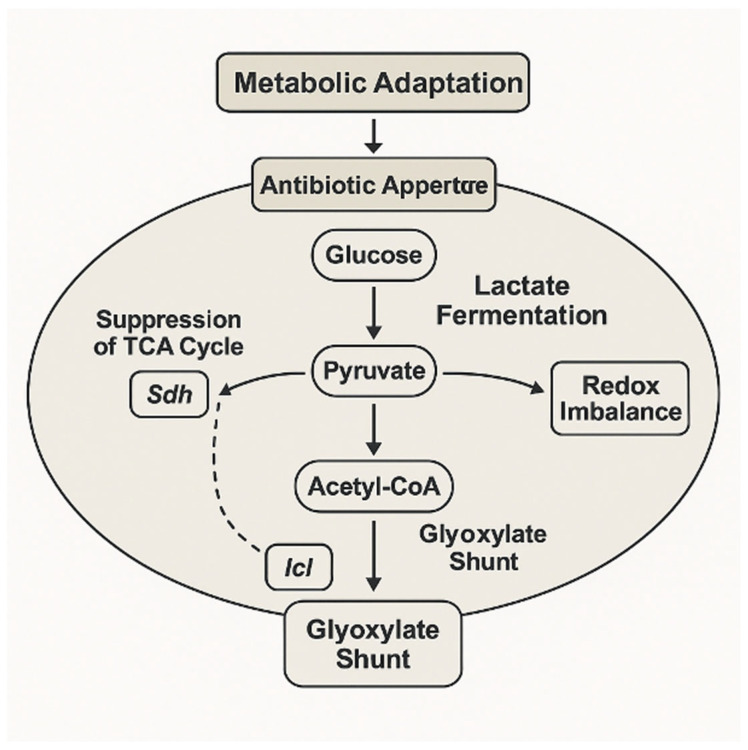
Schematic representation of bacterial metabolic adaptation in response to antibiotic exposure. Upon antibiotic pressure, glucose catabolism is rerouted from the tricarboxylic acid (TCA) cycle toward alternative pathways. Pyruvate, the central metabolic node, is diverted to lactate fermentation, contributing to redox imbalance and ATP generation via substrate-level phosphorylation. Concurrently, suppression of the TCA cycle—mediated by the reduced activity of enzymes such as succinate dehydrogenase (Sdh)—leads to the accumulation of acetyl-CoA. This promotes activation of the glyoxylate shunt, driven by isocitrate lyase (Icl), enabling carbon conservation and supporting bacterial persistence. These metabolic changes facilitate survival under antibiotic-induced stress by reducing reactive oxygen species production and preserving redox homeostasis. Image created using BioRender (accessed via web application, version as of May 2025), GraphPad Prism (version 10.1.2-2024), and PowerPoint (Microsoft 365, version 2404).

This metabolic rerouting, involving suppression of the TCA cycle and redirection of carbon flux through the glyoxylate shunt and fermentative pathways, exemplifies the adaptive strategy that supports bacterial survival under antibiotic stress (Figure 4). Enzymes such as succinate dehydrogenase and isocitrate lyase play key regulatory roles in this transition, mediating a shift toward carbon-efficient and redox-balanced states.

The main central carbon metabolic pathways implicated in bacterial tolerance and persistence during antibiotic exposure are summarized in Table 5.

### 4.3. Oxidative Stress and Redox Defenses: ROS–NADPH Balance

Many bactericidal antibiotics induce, in addition to their primary effect, an intracellular accumulation of reactive oxygen species (ROS), such as superoxide (O_2_^−^), hydrogen peroxide (H_2_O_2_), and hydroxyl radicals (OH•) [86].

This secondary oxidative effect arises from perturbations in central metabolism, particularly stimulation of cellular respiration and futile metabolic cycling, which elevate ROS production [7,86].

Intracellular accumulation of ROS in response to antibiotic treatment causes extensive oxidative damage to essential macromolecules, including DNA, proteins, and membrane lipids [6].

These molecular events compromise the structural and functional integrity of the bacterial cell, significantly contributing to the lethality of bactericidal antibiotics [6,7].

The contribution of ROS to antibiotic lethality is not uniform but depends on the antibiotic’s mechanism of action, the bacterial metabolic state, and the intrinsic capacity for redox detoxification [50].

Therefore, the secondary oxidative effect may amplify the cytotoxicity of certain antibiotics but does not constitute a universal mechanism across all pathophysiological contexts.

To mitigate antibiotic-induced oxidative stress, bacteria activate redox defense systems that include:

- the biosynthesis and regeneration of antioxidant molecules such as glutathione and thioredoxins, processes dependent on NADPH availability from the pentose phosphate pathway [87];
- the expression of antioxidant enzymes, including superoxide dismutase, catalase, and peroxiredoxins, which neutralize ROS [88]; and
- The activation of DNA repair systems, such as those mediated by RecA and polymerases IV and V, which correct ROS-induced genetic damage and support cell survival [89].

Redox responses activated under oxidative stress protect genomic integrity and modulate the cell cycle, promoting the formation of persister cells [90].

Maintaining the balance between NAD^+^ and NADPH is crucial to avoiding redox toxicity and sustaining the function of efflux pumps [90,91].

**Figure 5:**
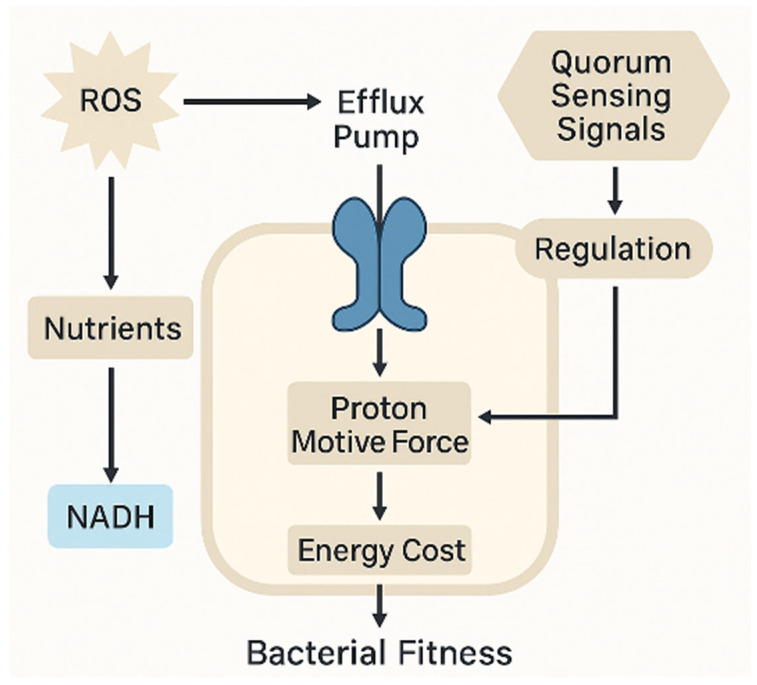
Energetic and regulatory constraints associated with efflux pump activity in bacterial antibiotic resistance. Efflux systems, regulated in part by quorum sensing signals, depend on the proton motive force (PMF) for active extrusion of toxic compounds, including reactive oxygen species (ROS). The generation of PMF requires metabolic energy, leading to an energy cost that may impact bacterial fitness. NADH produced from nutrient metabolism supports ROS detoxification and energy production. The energetic burden of maintaining efflux activity, particularly under antibiotic pressure, highlights the interplay between redox balance, signal integration, and bacterial survival strategies. Image created using BioRender (accessed via web application, version as of May 2025), GraphPad Prism (version 10.1.2-2024), and PowerPoint (Microsoft 365, version 2404).

The energetic burden imposed by efflux pump activity, regulated by quorum sensing and sustained by the proton motive force, exemplifies the trade-off between resistance mechanisms and bacterial fitness under oxidative and antibiotic stress (Figure 5). This integration of metabolic and regulatory inputs underscores the cost–benefit balance of non-genetic resistance strategies.

NADPH provides the reducing power necessary for antioxidant regeneration, while NAD^+^ is essential for catabolic reactions and the operation of efflux systems, which contribute to antibiotic tolerance and bacterial persistence [86,90,91].

### 4.4. Amino Acid Metabolism: Reserve, Signaling, and Persistence

Amino acids play essential roles in cellular metabolism, serving as energy reserves through their oxidation under nutrient-limiting conditions, acting as signaling molecules that modulate key metabolic pathways, and functioning as precursors for the biosynthesis of a wide array of bioactive compounds, including neurotransmitters, hormones, and nucleotides [92].

Under antibiotic pressure, bacteria profoundly modulate amino acid metabolism to adapt to stress and promote persistence.

In particular, arginine and proline activate the urea and carbamoyl phosphate pathways, contributing to the regulation of intracellular pH [93].

Tryptophan degradation leads to the production of indole, a signaling molecule that influences persister cell formation and the activation of toxin–antitoxin systems [94,95].

Finally, glutamine and glutamate support anaplerosis of the Krebs cycle and glutathione synthesis, sustaining energy production and defense against oxidative stress [96].

Biosynthetic pathways and amino acid transport systems are tightly regulated by metabolic circuits that respond to intracellular availability of ATP and NADPH [90,97].

These energy metabolites act as key signals, modulating enzymatic activity and gene expression to coordinate amino acid synthesis and import with the cell’s energetic and redox status [97].

Such regulation ensures efficient integration between amino acid metabolism and cellular bioenergetic demands [98].

Table 6 recapitulates the adaptive modulation of the TCA cycle and glyoxylate shunt as key survival mechanisms during antibiotic stress.

### 4.5. Membrane Biosynthesis and Lipid Adaptations

Bacterial membranes constitute a dynamic barrier whose composition and biophysical state significantly influence antibiotic uptake [99].

In Gram-negative bacteria, the outer membrane restricts the entry of many antibiotics, with permeability modulated by lipid composition and porin expression [100].

Membrane fluidity—determined by factors such as fatty acid composition and ambient temperature—profoundly affects the passive diffusion of lipophilic antibiotics across the phospholipid bilayer [101,102].

Alterations in fluidity serve as an adaptive strategy to modulate selective permeability [102].

Additionally, many antibiotics exert direct effects on the membrane by interacting with lipid components, inducing structural reorganizations that compromise barrier integrity and facilitate drug penetration [103].

In response to exposure to polycationic drugs, such as aminoglycosides and colistin, bacteria implement lipid composition adjustments to mitigate the toxic effects of these antibiotics [104].

These modifications include the increased incorporation of branched and saturated fatty acids, which reduce membrane fluidity and limit antibiotic penetration; the incorporation of amino groups such as 4-amino-4-deoxy-L-arabinose into LPS, thereby decreasing the net negative charge of the outer membrane and reducing affinity for cationic antibiotics; and the accumulation of triacylglycerols in intracellular vesicles, which may serve as energy reserves and contribute to antibiotic tolerance [104,105,106].

Bacterial membrane adaptations, regulated by the two-component systems PhoPQ and PmrAB, represent critical strategies for survival in hostile environments and under antibiotic pressure [107].

These systems coordinate the synthesis of lipid enzymes and transporters that alter membrane composition, influencing permeability and the electrochemical potential [108].

Such modifications can impair antibiotic uptake efficiency, compromising their effectiveness [108].

## 5. Interaction with the Host and Microbiota: Ecological Dynamics, Adaptive Niches, and Immunometabolic Signals

The metabolic reprogramming of bacterial pathogens does not occur in a biochemical vacuum but rather unfolds within complex and dynamic ecosystems dominated by interactions among the bacterium, the host, and the commensal microbiota [109,110].

In this context, antibiotics and environmental signals act both as selective pressures and as stimuli for metabolic restructuring in bacterial pathogens, promoting the emergence of adaptive phenotypes closely linked to survival within host microenvironments [111].

These stimuli induce changes in bacterial metabolic and morphological pathways, facilitating adaptation to hostile conditions and contributing to infection persistence [112].

### 5.1. Antibiotics and Ecological Disruption of the Microbiota

While antibiotic therapy is aimed at pathogen elimination, it induces profound alterations in the composition and function of the commensal microbiota, disrupting intestinal ecological balance and potentially predisposing the host to dysbiosis and opportunistic infections [113].

Antibiotic-induced disruption of the microbiota leads to the creation of vacant ecological niches and a reduction in interbacterial competition—conditions that favor the clonal expansion of opportunistic bacterial strains with high metabolic plasticity [114].

These microorganisms, often antibiotic-resistant, can effectively colonize the compromised intestinal ecosystem, further altering microbial composition and increasing the risk of dysbiosis and opportunistic infections [115].

Several intestinal pathogens, including *Clostridioides difficile*, *Klebsiella pneumoniae*, and *Enterococcus faecium*, exploit metabolites released from the lysed commensal microbiota—such as succinate, sialate, and ethanolamine—to activate invasive metabolic programs [116].

These metabolites serve as both signals and substrates that promote biofilm formation and bacterial persistence, facilitating host colonization and increasing the risk of recurrent infections [117].

Table 7 summarizes the ecological and metabolic consequences of antibiotic-induced microbiota disruption that favor colonization by metabolically adapted pathogens.

### 5.2. Anatomical Niches and Local Metabolism: The Role of Biofilms

Anatomical niches characterized by poor perfusion, altered pH, or hypoxic conditions—such as cardiac valves, fibrotic lungs, and urinary tracts—provide microenvironments conducive to biofilm formation, wherein microbial communities are encapsulated in a self-produced extracellular matrix [118].

These environmental conditions promote bacterial adhesion, matrix component production, and microbial persistence, contributing to antimicrobial resistance and chronic infection development [119].

Within biofilms, bacterial cells exhibit significant physiological heterogeneity, marked by reduced respiratory activity and a predominance of fermentative metabolic pathways [120,121].

Specific metabolic pathways are activated, including those of glycogen, acetate, and glycerol—adaptations that support survival under environmental stress. In parallel, there is an accumulation of extracellular polymeric substances (EPS) and redox-active proteins, which enhance cellular protection and structural stability of the biofilm [120,121].

These conditions promote persistent and stratified metabolic rewiring, with differentiated subpopulations exhibiting high levels of tolerance [122].

Moreover, the biofilm extracellular matrix functions as a physical barrier, impeding antibiotic penetration and protecting bacterial cells from antimicrobial agents [122].

### 5.3. Host Immunometabolites as Environmental Signals

During infection, the host releases a variety of bioactive metabolites—including lactic acid, itaconate, succinate, nitric oxide, and reactive oxygen species—that act as immunometabolites, modulating immune responses and influencing pathogen metabolic adaptation [123].

Bacteria detect host-derived immunometabolic signals such as itaconate and nitric oxide through specific metabolic sensors, inducing adaptive responses that enhance survival and persistence [124].

Itaconate, produced by activated macrophages, inhibits isocitrate lyase, thereby impairing the glyoxylate cycle and forcing a reallocation of carbon flux [124].

Concurrently, nitric oxide inhibits bacterial respiration, prompting activation of alternative anaerobic pathways [125].

Bacterial signaling systems, including GacS/GacA and PhoPQ, mediate these adaptive responses by regulating the expression of genes involved in virulence and biofilm formation [126,127].

These metabolic responses should not be viewed solely as defensive mechanisms but rather as a form of inter-kingdom communication, in which host-derived metabolites directly modulate gene expression, virulence, and metabolic adaptation in pathogens.

Table 8 summarizes the effects of key host-derived immunometabolites on bacterial metabolic pathways and persistence strategies.

### 5.4. Quorum Sensing, Nutrient Sensing, and Intra-Microbial Cooperation

Under antibiotic pressure, bacterial metabolism is regulated by quorum sensing (QS) systems, which integrate signals related to cell density and nutrient availability [10].

Quorum sensing systems finely modulate gene expression to optimize survival in hostile environments, coordinating collective processes such as biofilm formation, secretion of virulence factors, and metabolic adaptation to nutrient availability [128].

In *Pseudomonas aeruginosa*, activation of the Las and Rhl quorum sensing systems directly modulates the expression of efflux pumps and lipid biosynthesis in response to cell density and the presence of antibiotics [129].

By integrating environmental signals through quorum sensing pathways, *P. aeruginosa* finely tunes its metabolism and virulence expression, efficiently adapting to hostile conditions and promoting survival in microenvironments characterized by nutritional, immune, or antimicrobial stress [130].

In parallel, nutrient sensing mediated by the PTS system and the global regulator CodY enables bacteria to discriminate between favorable and stressful environmental conditions, determining whether to activate growth programs or enter dormancy [131].

Through the integration of intracellular metabolic signals and environmental stimuli, regulatory systems such as PTS and CodY finely modulate gene expression, enabling bacterial metabolic and transcriptional adaptation to dynamic and hostile microenvironments, thereby optimizing survival and persistence [131].

### 5.5. Antagonistic Interaction with the Microbiota: The Role of Toxic Metabolites

Metabolites derived from the intestinal microbiota—including short-chain fatty acids, secondary bile acids, and antimicrobial peptides—exert selective pressures on the microbial ecosystem [132,133].

These microbiota-derived metabolites, such as short-chain fatty acids and secondary bile acids, modulate bacterial gene expression and metabolic pathways, exerting selective pressures that inhibit pathogen proliferation and promote metabolic adaptations conducive to survival in dynamic and competitive environments [134].

For example:

- Butyrate inhibits the proliferation of *Salmonella Typhimurium* by acidifying the intracellular environment and modulating virulence gene expression [135]. However, *Clostridioides difficile* can utilize butyrate as an energy source, exploiting its presence to sustain growth in nutrient-limited conditions [136].
- Primary bile acids, such as taurocholic acid, promote *C. difficile* spore germination, facilitating infection [137]. In contrast, secondary bile acids such as deoxycholic acid (DCA) and lithocholic acid (LCA) inhibit germination and vegetative growth of *C. difficile*, contributing to colonization resistance [138].

These chemical signals in the intestinal microenvironment—including microbial metabolites and immune-derived molecules—can profoundly reshape the metabolic physiology of pathogens, promoting phenotypic states of persistence or hypervirulence depending on the nature and combination of environmental cues [139].

## 6. Therapeutic Implications: Targeted Strategies, Metabolic Targets, and Combined Approaches

The recognition of metabolic reprogramming as a key factor in determining antibiotic tolerance and in the transition toward genetic resistance opens new avenues for the development of innovative antimicrobial therapies [111].

Targeted interventions aimed at modulating bacterial metabolic pathways could enhance the efficacy of existing antibiotics and prevent the emergence of resistance [140].

Selective modulation of bacterial metabolic circuits represents a novel therapeutic opportunity to restore drug sensitivity, eradicate persistent populations, and hinder the emergence of antibiotic-resistant strains [140].

This section explores three emerging therapeutic strategies: direct targeting of metabolic enzymes, co-administration of metabolic modulators, and development of metabolically selective antibiotics.

### 6.1. Targeting Key Enzymes of Bacterial Metabolism

Several enzymes of central metabolism, absent or poorly represented in eukaryotic cells, constitute selective targets for the development of new antibacterial agents.

Among these, isocitrate lyase (ICL), a key enzyme of the glyoxylate cycle, is essential for the survival of *Mycobacterium tuberculosis* during latency, making it a promising target for antitubercular therapies [83].

In addition, glutamate dehydrogenase and enzymes involved in glutathione biosynthesis play a crucial role in maintaining bacterial redox homeostasis, influencing antibiotic response and representing potential targets for innovative therapeutic strategies [141].

Inhibitors of these enzymes may potentiate antibiotic activity or reduce MIC thresholds in clinically refractory settings.

Table 9 identifies metabolic enzymes that represent promising targets for enhancing antibiotic efficacy through selective inhibition, with potential application in latency-phase pathogens and redox-based tolerance.

### 6.2. Metabolite Adjuvants: Synergistic Modulation of Response

The targeted use of metabolites or cofactors represents a promising strategy to enhance antibiotic efficacy by shifting bacterial metabolism toward more vulnerable states. Notable examples include:

- Administration of α-ketoglutarate: enhances aminoglycoside efficacy by increasing ROS production, thereby contributing to bacterial lethality [142].
- Use of fructose and mannose: improves aminoglycoside uptake by boosting glycolysis and the proton motive force, demonstrating efficacy against persister cells [143].
- Application of nitric oxide (NO) donors: induces oxidative and nitrosative stress, increasing the lethality of ciprofloxacin in *E. coli* [144].

These metabolic strategies have shown efficacy in both in vitro and in vivo models, including against persister cells, underscoring the therapeutic potential of metabolism-based approaches to enhance antibiotic activity and counteract tolerance.

As detailed in Table 10, metabolite adjuvants can modulate bacterial metabolism to enhance the efficacy of conventional antibiotics through diverse mechanisms, including redox modulation and uptake facilitation.

### 6.3. Antimetabolites and Hybrid Drugs: Multilevel Interference

Fosfomycin, a broad-spectrum antibiotic known for its covalent inhibition of MurA—catalyst of the first step in peptidoglycan biosynthesis—has attracted renewed interest as a potential “tool drug” for identifying bacterial metabolic vulnerabilities [145].

Although its use in this context has yet to be extensively validated, fosfomycin’s specificity makes it a promising candidate for studies aimed at uncovering metabolic weaknesses in bacterial pathogens.

Antibiotics that function as antimetabolites—by inhibiting key metabolic processes such as folate biosynthesis, NAD^+^ production, and oxidative phosphorylation—can enhance the efficacy of conventional antibiotics [146].

For example, the combination of sulfonamides and trimethoprim produces sequential inhibition within the folate synthesis pathway, resulting in a synergistic bactericidal effect [146].

Similarly, isoniazid, through the formation of complexes with NAD^+^ and NADP^+^, disrupts mycolic acid synthesis in mycobacteria, thereby potentiating the activity of other antimycobacterial agents [147].

Moreover, the inhibition of oxidative phosphorylation—as observed with bedaquiline—compromises ATP production in pathogens, increasing their susceptibility to antibiotics [148].

Although no hybrid drugs have yet been documented that combine an oxazolidinone with ribonuclease activity, the design of multifunctional antimicrobial agents is an area of growing interest [149].

Such strategies aim to enhance therapeutic efficacy by integrating antimicrobial activity with additional biological functions, such as inhibition of specific bacterial metabolic processes.

### 6.4. Combination Therapies and Forced Reprogramming

The therapeutic strategy of combining metabolites with antibiotics aims to modulate bacterial metabolism to enhance susceptibility to antimicrobial agents.

Although specific evidence on the combination of fosfomycin and glycine in reducing *Enterobacter* resistance is limited, studies have shown that inactivation of the tricarboxylic acid (TCA) cycle in *Staphylococcus aureus* leads to increased formation of persister cells and reduced antibiotic sensitivity [42].

These findings suggest that modulation of central metabolism can influence antibiotic efficacy, offering new perspectives for the treatment of bacterial infections.

Such therapies offer a key advantage: their synergistic effect is less susceptible to genetic resistance, as it targets highly conserved metabolic circuits.

### 6.5. Metabolite-Guided Diagnostics and Therapy: Precision Perspectives

Understanding metabolic rewiring paves the way for strategies of precision antimicrobial therapy based on in vivo pathogen metabolic profiling.

Imaging of D_2_O metabolism at the single-cell level via Stimulated Raman Scattering (SRS) represents a methodological advance that enables rapid determination of antimicrobial susceptibility, allowing the real-time identification of metabolically tolerant bacterial phenotypes [150].

Early identification of the metabolic state of bacterial pathogens—whether dormant, oxidative, glycolytic, or fermentative—can inform the selection of targeted combinations of antibiotics and metabolites, enabling a personalized approach that optimizes treatment efficacy [19].

Modulation of bacterial metabolism through the addition of specific exogenous metabolites has been shown to restore antibiotic sensitivity in tolerant strains, underscoring the therapeutic potential of metabolism-based strategies to improve clinical outcomes [151].

## 7. Limitations of the Current Literature and Future Perspectives

Despite growing interest in the role of metabolic rewiring in bacterial survival under antibiotic stress, significant theoretical, experimental, and translational gaps remain.

Key limitations include: (1) the lack of an integrated molecular model describing the involved metabolic interactions; (2) the absence of standardized experimental protocols, which hinders cross-study comparability; (3) limited translation of in vitro findings to clinical settings due to confounding factors such as biofilm formation and the presence of persister cells; and (4) the underutilization of omics technologies, which—if properly integrated—could provide deeper insights into bacterial responses and guide the development of personalized therapeutic strategies.

### 7.1. Incomplete Models and Conceptual Fragmentation

The current scientific literature on bacterial metabolic rewiring in response to antibiotic stress is characterized by several limitations.

Many studies focus on individual metabolic pathways in isolation, often overlooking the systemic interconnectivity of metabolic networks [140,152,153].

Moreover, bacterial metabolic rewiring is frequently portrayed as a binary process—either active or inactive—without accounting for its temporal dynamics, the metabolic heterogeneity within bacterial populations, or the influence of environmental and immunological factors [154].

Recent evidence, however, indicates that bacterial metabolism is highly dynamic and subject to temporal variation in response to external stimuli [140,154].

Within a single bacterial population, metabolically distinct subpopulations may coexist, significantly influencing the overall response to antibiotics [155].

Additionally, the host microenvironment and immune interactions play a critical role in modulating bacterial metabolism and, consequently, antibiotic efficacy [156].

These oversimplifications limit a deeper understanding of the metabolic mechanisms underlying antibiotic tolerance and resistance.

A unifying theory is still lacking—one that frames rewiring as an eco-evolutionary phenomenon capable of explaining the transition from metabolic tolerance to stable genetic resistance, integrating biochemistry, microbial ecology, and complex systems theory.

### 7.2. Methodological Limitations and Experimental Biases

Commonly used in vitro models for studying bacterial metabolism and antibiotic responses exhibit several limitations that compromise their clinical translatability.

First, the use of laboratory-adapted bacterial strains may not accurately reflect the physiology of clinical pathogens [157].

Second, artificial environmental conditions—characterized by high oxygenation, constant pH, and absence of host signals—fail to replicate the physiological milieu encountered by bacteria during infections [158].

Furthermore, the use of non-standardized antibiotic concentrations can affect the selection of resistant strains and the assessment of therapeutic efficacy [157,158].

Finally, the lack of dynamic metabolic measurements—such as isotopic turnover and single-cell metabolomics—limits our understanding of temporal metabolic responses and intra-population heterogeneity [157,158].

These limitations underscore the need for more representative experimental models and advanced analytical technologies to improve the clinical relevance of in vitro findings.

As a result, outcomes are often difficult to generalize and apply in clinical settings. Additionally, many studies fail to distinguish between tolerance, persister cells, and resistance, contributing to conceptual confusion.

### 7.3. Weak Link Between Molecular Data and Clinical Outcomes

Despite the growing body of molecular data on bacterial metabolic rewiring in response to antibiotics, correlations between these metabolic changes and clinically relevant parameters—such as bacterial clearance time, treatment failure unrelated to genetic resistance, post-therapy recurrence, and the in vivo selection of resistant mutants—remain poorly investigated [159,160].

A deeper understanding of these associations is essential to translate metabolic insights into effective, personalized therapeutic strategies.

The lack of clinical trials and longitudinal omics approaches hampers efforts to determine whether metabolic rewiring is a laboratory epiphenomenon or a genuine determinant of infectious disease prognosis.

### 7.4. Future Perspectives: Toward an Integrated and Translational Vision

To address these gaps, we propose a research agenda organized around five key directives:

- Development of integrated multi-omics models, combining transcriptomics, metabolomics, fluxomics, and metabolic imaging to define real-time functional maps.
- Standardization of in vitro and in vivo models, using host-mimicking environments (e.g., organoids, immunocompetent murine models, artificial body fluids).
- Diagnostic metabolic profiling in patients, based on mass spectrometry from clinical samples, with correlation to tolerance/resistance phenotypes.
- Metabolite-guided clinical trials, testing combination approaches involving antibiotics and metabolic modulators.
- Development of smart drugs targeting conserved bacterial pathways absent in mammals (e.g., isocitrate lyase, NAD^+^ regeneration, bacterial-specific redox systems).

### 7.5. Proposal for a Conceptual Framework: Metabolic Plasticity as an Evolutionary Axis

In light of current evidence, we propose a conceptual model in which metabolic plasticity serves as an intermediate axis between the tolerant phenotype and the resistant genotype. Metabolic rewiring, induced by antibiotics or ecological stressors, creates selective niches where adaptive mutations accumulate. This “metabolic–evolutionary” axis provides a framework for understanding the temporal transition from transient survival to stable resistance, integrating:

- antibiotic-dependent fitness reduction;
- positive selection of secondary mutations; and
- intra-population metabolic heterogeneity as an evolutionary reservoir.

## 8. Conclusions

In recent years, advances in bacterial physiology have brought to light a previously underappreciated but crucial aspect: the ability of pathogens to respond to antibiotic pressure through profound and coordinated metabolic reprogramming [111,140].

This phenomenon, distinct from classical mechanisms of genetic resistance, allows bacteria to enter reversible phenotypic states of tolerance, persistence, and ecological adaptation, thereby significantly reducing antibiotic efficacy even in the absence of mutations [161].

Accumulated evidence demonstrates that numerous metabolic pathways—from glycolysis and respiration to amino acid metabolism and lipid biosynthesis—are modulated in response to various antibiotics in a pathway-specific and function-dependent manner.

These adaptations are not passive: they are active, regulated, and highly plastic responses capable of reshaping cellular fate and promoting microenvironments conducive to survival.

The clinical consequences are substantial. Therapeutic failure during infections caused by strains that are susceptible in vitro, recurrence, intracellular persistence, and the evolutionary selection of resistant mutants are often attributable to these adaptive metabolic states. Therefore, metabolic rewiring should not be viewed merely as a biological curiosity, but as an emerging paradigm of non-genetic resistance that must be recognized, investigated, and addressed systematically.

From a therapeutic perspective, understanding these mechanisms opens new avenues: the development of drugs targeting bacterial metabolic enzymes, the use of co-treatments with sensitizing metabolites, real-time diagnosis guided by metabolic profiling, and the design of personalized strategies based on the pathogen’s bioenergetic state. In this light, a paradigm shift is necessary: from direct attack on molecular targets to a more sophisticated and dynamic approach that also considers the pathogen’s metabolic network, host microenvironment, and microbial interactions.

Finally, this review advocates for recognizing metabolic rewiring not as an epiphenomenon but as an active evolutionary axis—a bridge between ecological adaptation and genetic selection. Integrating this perspective into preclinical models, clinical trials, and everyday infectious disease practice represents the next frontier in the fight against resistant infections.

## Figures and Tables

**Table 1:** Mechanistic basis of metabolic rewiring in response to antibiotic stress.

Mechanism	Description	References
Glycolysis and Krebs Cycle Remodeling	Reconfiguration of central carbon metabolism to redirect energy flow and optimize survival	[12,13,14]
Glyoxylate Shunt Activation	Diversion of carbon flux through secondary pathways to reduce ROS generation	[5,7,23]
Lipid and Amino Acid Metabolism Shifts	Reprogramming of biosynthetic routes for adaptive stress responses	[12,13,14]
Modulation of Membrane Composition	Alteration in lipid profile to affect antibiotic permeability	[25,26]
Suppression of Respiration and ROS	Downregulation of oxidative metabolism to limit bactericidal ROS activity	[6,27]
Growth Arrest and Metabolic Slowdown	Temporal dormancy to outlast antibiotic action	[22,28]

ROS: reactive oxygen species.

**Table 2:** Distinguishing features of metabolic rewiring versus genetic resistance.

Feature	Metabolic Rewiring	Genetic Resistance	References
Nature	Phenotypic and reversible	Genotypic and heritable	[11,15]
Genetic Modification	Absent	Present (mutations, gene acquisition)	[15,16,17]
Duration	Transient	Persistent	[11,15]
MIC Change	Usually unchanged	Increased MIC	[18,19]
Mechanisms	Redox modulation, metabolic rerouting	Enzymatic inactivation, efflux, target alteration	[5,6,7,16,17,23]
Evolutionary Role	Potential precursor to resistance	End-point of selection	[24,31,32]

MIC: minimum inhibitory concentration.

**Table 3:** Metabolic adaptations to different classes of antibiotics.

Antibiotic Class	Target Mechanism	Metabolic Response	References
β-lactams	Inhibit PBPs and peptidoglycan synthesis	Upregulation of peptidoglycan precursor pathways, glyoxylate cycle activation, fermentative shift	[6,35,36,37,38,39,40,41,42,43]
Aminoglycosides	Bind ribosomal subunits, require proton motive force	Fermentative metabolism, reduced respiration, increased glutathione/NADPH pathway activity	[6,7,12,44,45,46]
Quinolones	Inhibit DNA gyrase/topoisomerase, induce ROS	Suppression of TCA cycle, activation of glyoxylate cycle, reduced ROS via altered redox flux	[4,6,7,47,48,49,50,51,52]
Glycopeptides	Bind D-Ala-D-Ala, block peptidoglycan cross-linking	Metabolic quiescence, reorientation of carbon metabolism, phosphoenolpyruvate-pyruvate-oxaloacetate flux	[12,53,54,55,56]
Polymyxins	Disrupt outer membrane via lipid interaction	Lipid A modification (L-Ara4N, pEtN), altered lipid biosynthesis, activation of two-component systems	[57,58,59,60,61,62,63]
Antimetabolites	Block nucleotide or cell wall precursor biosynthesis	Uptake of purines, derepression of purine synthesis, activation of pentose phosphate pathway	[64,65,66,67,68]

PBPs: penicillin-binding proteins; TCA: tricarboxylic acid; ROS: reactive oxygen species; L-Ara4N: 4-amino-4-deoxy-L-arabinose; pEtN: phosphoethanolamine.

**Table 4:** Glycolysis and fermentative reprogramming under antibiotic stress.

Metabolic Node	Adaptive Change	Functional Outcome	References
Pyruvate Flux	Shift to lactate/acetate fermentation	NAD^+^ regeneration, ROS reduction, ATP from substrate-level phosphorylation	[70,71,72,73]
Respiration	Suppression under antibiotic stress	Decreased ROS production	[70,71,72]
NAD^+^/NADH Balance	Restoration via fermentative pathways	Maintains redox homeostasis	[70,71,72,73]
Regulatory Systems	Activation of SrrAB, NreBC	Controls fermentative gene expression	[74,75]

NAD^+^: nicotinamide adenine dinucleotide; ROS: reactive oxygen species.

**Table 5:** Role of central carbon metabolism in antibiotic tolerance and persistence.

Pathway	Antibiotic Association	Functional Outcome	References
Glyoxylate Cycle	β-lactams, Quinolones	Carbon conservation, reduced ROS, support for cell wall regeneration	[5,6,7,23,40,41,51,52]
Tricarboxylic Acid (TCA) Cycle	β-lactams, Quinolones	Downregulation lowers ROS and slows growth	[6,42,43,50]
Fermentation Pathways	β-lactams, Aminoglycosides	Decreased oxidative stress, energy conservation, growth rate modulation	[6,7,12,42,43]
Pentose Phosphate Pathway	Fosfomycin, Antimetabolites	NADPH production, support for anabolic metabolism	[66,67,68]
Anaplerotic Flux (PEP–OAA Axis)	Glycopeptides	Maintenance of precursor supply for cell wall biosynthesis	[56]

ROS: reactive oxygen species; PEP: phosphoenolpyruvate; OAA: oxaloacetate.

**Table 6:** TCA cycle modulation and glyoxylate shunt activation in antibiotic adaptation.

Pathway Component	Stress-Driven Change	Adaptive Benefit	References
Aconitase, SDH, α-KGDH	Suppressed due to Fe–S cluster sensitivity to ROS	Reduces oxidative damage	[77,78,79]
TCA Cycle	Downregulated in hostile environments	Limits ROS, slows metabolism	[78,79]
Glyoxylate Shunt	Activated via isocitrate lyase and malate synthase	Carbon conservation, evasion of CO_2_ loss	[80,81,82,83,84,85]
*Mycobacterium tuberculosis*	Relies on glyoxylate shunt during latency	Supports survival under immune and antibiotic stress	[82,83,84,85]

SDH: succinate dehydrogenase; α-KGDH: alpha-ketoglutarate dehydrogenase; CO_2_: carbon dioxide.

**Table 7:** Ecological and metabolic impact of antibiotics on the gut microbiota.

Ecological Effect	Mechanism	Pathogen Adaptation Strategy	References
Microbiota Disruption	Loss of commensal competition and niche availability	Clonal expansion of metabolically plastic opportunists	[113,114,115]
Metabolite Release	Lysis of microbiota releases substrates (succinate, sialate, ethanolamine)	Use of host-derived substrates to activate virulence and persistence	[116,117]
Dysbiosis-Driven Colonization	Depletion of colonization resistance factors	Biofilm formation and recurrent infection by *C. difficile*, *K. pneumoniae*, *E. faecium*	[113,114,115,116,117]

**Table 8:** Host-derived immunometabolites and bacterial metabolic responses.

Immunometabolite	Bacterial Sensor or Effect	Metabolic Response or Impact	References
Itaconate	Inhibits isocitrate lyase	Blocks glyoxylate cycle, alters carbon flux	[124]
Nitric Oxide (NO)	Inhibits respiratory chain	Induces anaerobic and alternative metabolism	[125]
Lactic Acid	Environmental acidification	Stress signal influencing gene regulation	[123]
Succinate	Signal molecule for inflammation and bacterial energy use	Supports anaerobic respiration and stress adaptation	[123,124]
Regulatory Pathways	GacS/GacA, PhoPQ	Coordinate metabolic and virulence responses to host signals	[126,127]

**Table 9:** Enzymatic targets for metabolism-based antibacterial therapy.

Enzyme Target	Function in Bacteria	Therapeutic Rationale	References
Isocitrate Lyase (ICL)	Glyoxylate cycle enzyme critical in latency	Target in *M. tuberculosis* for latent phase survival	[83]
Glutamate Dehydrogenase	Redox balance and nitrogen metabolism	Target to disrupt redox homeostasis	[141]
Glutathione Biosynthetic Enzymes	Maintain intracellular ROS detoxification	Inhibition sensitizes bacteria to oxidative antibiotics	[141]

ROS: Reactive Oxygen Species.

**Table 10:** Metabolic adjuvants enhancing antibiotic efficacy.

Adjuvant Agent	Mechanism of Action	Potentiated Antibiotic(s)	References
α-Ketoglutarate	Promotes ROS accumulation	Aminoglycosides	[142]
Fructose, Mannose	Boost glycolysis and proton motive force	Aminoglycosides (e.g., gentamicin)	[143]
Nitric Oxide (NO) Donors	Induce oxidative/nitrosative stress	Ciprofloxacin	[144]
Sulfonamides + Trimethoprim	Sequential folate pathway inhibition	Broad-spectrum bactericidal synergy	[144]
Isoniazid	Disrupts NAD^+^-dependent mycolic acid synthesis in mycobacteria	Antimycobacterial regimens	[143]
